# Effectiveness of alcohol brief intervention delivered by community pharmacists: two-arm randomised controlled trial

**DOI:** 10.1186/1940-0640-8-S1-A21

**Published:** 2013-09-04

**Authors:** Ranjita Dhital, Cate Whittlesea, Ian J Norman, Trevor Murrells, Jim McCambridge

**Affiliations:** 1 King’s College London, Florence Nightingale School of Nursing and Midwifery, London, UK; 2 King’s College London, King’s Health Partners, Pharmaceutical Science Clinical Academic Group, Institute of Pharmaceutical Science, London, UK; 3 London School of Hygiene & Tropical Medicine, Department of Social and Environmental Health Research, London, UK

## 

The UK Department of Health’s aim is to involve community pharmacists in the delivery of alcohol brief intervention (BI). This possibility extends the settings in which BIs have been delivered and has attracted international attention. The objective of this study was to assess the effectiveness of BI delivered by community pharmacists in a randomised controlled trial (RCT) in London. A two-arm RCT was conducted to determine the effectiveness of BI delivered by community pharmacists. Pharmacists and their support staff were trained in the trial procedures. Pharmacy support staff (N = 23) approached and informed customers about the study, to support formal recruitment by the community pharmacist (N = 17). Eligible and consenting pharmacy customers (aged≥18 years) at the 16 community pharmacies were randomised in equal numbers to either BI delivered by a community pharmacist or a non-intervention control condition, conducted in the pharmacy consultation room. The intervention was a brief motivational discussion of approximately 10 minutes duration. Participants randomised to the control arm were given an alcohol information leaflet with no opportunity for discussion. At 3-month follow up, alcohol consumption and related problems were assessed with the Alcohol Use Disorders Identification Test (AUDIT) administered by telephone. Of the 1440 customers approached, 541 (38%) consented to participate. Of those who consented, 409 were identified as hazardous/harmful drinkers (AUDIT 8-19), 94 as low risk drinkers (AUDIT≤7), and 38 as possibly dependent drinkers (AUDIT≥20), with the latter two groups excluded from the trial. The 409 trial participants were followed up at three months (follow-up rate ≥80%). Data analysis is under way and the preliminary main trial results are presented here. This is the first presentation of trial outcomes from this internationally significant study.

